# Ultra-thin Glass Film Coated with Graphene: A New Material for Spontaneous Emission Enhancement of Quantum Emitter

**DOI:** 10.1007/s40820-015-0037-5

**Published:** 2015-04-04

**Authors:** Lu Sun, Chun Jiang

**Affiliations:** State Key Laboratory of Advanced Optical Communication System and Networks, Shanghai Jiao Tong University, Shanghai, 200240 People’s Republic of China

**Keywords:** Graphene surface plasmons, Graphene plasmonics, Quantum electrodynamics, Superradiance, Subwavelength structures

## Abstract

We propose an ultra-thin glass film coated with graphene as a new kind of surrounding material which can greatly enhance spontaneous emission rate (SER) of dipole emitter embedded in it. With properly designed parameters, numerical results show that SER-enhanced factors as high as 1.286 × 10^6^ can be achieved. The influences of glass film thickness and chemical potential/doping level of graphene on spontaneous emission enhancement are also studied in this paper. A comparison is made between graphene and other coating materials such as gold and silver to see their performances in SER enhancement.

## Introduction

Since the discovery of Purcell effect in 1946, a lot of research interests have been focused on the radiation environment of an emitter [1]. Apart from manipulating the emitter, spontaneous emission rate (SER) can also be enhanced or suppressed by its surrounding materials, including metamaterials such as photonic crystals and metal–insulator–metal plasmonic structures [2–5]. Owing to the small mode volume, the SER-enhanced factors obtained in metamaterials are usually several orders higher than those in natural materials. However, losses are remarkable in these small mode volumes, e.g., energy will leak into free space from planar photonic crystals and metal resistance will cause dissipations in plasmonic structures. So the SER-enhanced factors are generally limited to the order of 10^2^–10^3^ [2–5].

Graphene, a monolayer of carbon atoms arranged in a two-dimensional hexagonal lattice, has attracted great research interests since it was first isolated from bulk graphite in 2004 [6]. The surface plasmon (SP) of doped graphene has lower losses compared to that of traditional plasmonic materials [7]. Its electromagnetic fields are highly confined to the graphene monolayer [8]. Therefore, graphene has become a promising material for SER enhancement.

Due to all the advantages mentioned above, graphene materials such as single-layer sheet, nanoribbon, and nanodisk have already been investigated for strong light-matter interaction [8–11]. However, since graphene is an atomically thick sheet of carbon, quantum emitter cannot be embedded into it and the electromagnetic fields radiated by the emitter are poorly confined. Thus, we consider constructing a new type of metamaterial made of graphene to improve it. In our previously reported work, we designed a double-layer graphene waveguide which greatly enhances the spontaneous emission of quantum emitter placed in it [12]. However, it requires static gate voltages to control the chemical potential of graphene, which makes it an active device. The electrodes attached to the graphene layers would bring great complexity to the fabrication processes.

In this paper, we propose a new kind of metamaterial consisting of an ultra-thin glass film coated with doped graphene monolayers. The chemical potential of graphene is manipulated through chemical doping instead of electrical tuning, so it is more like a passive material than an active device. Since mid-infrared (MIR) light sources are essential for applications that include free-space communication, chemical and biomolecular sensing, and infrared spectroscopy [13, 14], we focus on the wavelength range from 3000 to 6000 nm. With proper doping level, interband transitions are forbidden in graphene for this regime and SPs sustain lower losses [7]. Quantum emitters embedded in the glass will experience strong radiation reaction due to the graphene coating. Using finite-difference time-domain (FDTD) techniques, we prove that SER of quantum emitter has been greatly increased in the proposed material. Our design can make single-photon MIR source far more efficient in radiation, which could find applications in single-plasmon devices and quantum plasmonics [8]. Furthermore, stimulated emission is related to spontaneous emission by the boson nature of photon [15]. Therefore, it will be enhanced either due to the Purcell effect, and the radiation of conventional MIR light sources will also be improved by our material.

## Theoretical Analysis on the Proposed Metamaterial

Quantum emitter, generally small in size, can be approximated by an electric dipole in physical models. The SER-enhanced factor or Purcell factor can be calculated by [16]:

1 $$ \frac{\gamma }{{\gamma_{0} }} = \frac{{{\mathbf{n}}_{{\mathbf{p}}} \cdot {\text{Im[}}{\mathbf{G}}({\mathbf{r}}_{ 0} ,{\mathbf{r}}_{ 0} ,\omega ) ]\cdot {\mathbf{n}}_{{\mathbf{p}}} }}{{{\mathbf{n}}_{{\mathbf{p}}} \cdot {\text{Im[}}{\mathbf{G}}_{ 0} ({\mathbf{r}}_{ 0} ,{\mathbf{r}}_{ 0} ,\omega ) ]\cdot {\mathbf{n}}_{{\mathbf{p}}} }} $$

where *γ* is the SER of electric dipole in surrounding material, *γ*_0_ is the one in free space, **n**_**p**_ is the unit orientation vector of dipole moment **p**, **G** (**r**_0_, **r**_0_, *ω*) is the dyadic Green function in inhomogeneous environment, **G**_0_ (**r**_0_, **r**_0_, *ω*) is the dyadic Green function in free space, **r**_0_ and *ω* are the location and oscillation frequency of an emitter. The imaginary part of **G** (**r**, **r**, *ω*) is related to the quantized electromagnetic field by [16]:

2 $$ {\text{Im[}}{\mathbf{G}}({\mathbf{r}},{\mathbf{r}},\omega ) ]= \frac{{\pi c^{ 2} }}{2\omega }\sum\limits_{\mathbf{k}} {{\mathbf{u}}_{\mathbf{k}}^{ *} ({\mathbf{r}} ,\omega_{\mathbf{k}} ){\mathbf{u}}_{\mathbf{k}} ({\mathbf{r}},\omega_{\mathbf{k}} )\delta (\omega - \omega_{\mathbf{k}} )} $$

where **u**_**k**_ are the normal modes satisfying.

3 $$ \int\limits_{V} {{\mathbf{u}}_{\mathbf{k}} ({\mathbf{r},}\omega_{\mathbf{k}} )\cdot {\mathbf{u}}_{\mathbf{k'}}^{ *} ( {\mathbf{r},}\omega_{\mathbf{k'}} )d^{ 3} {\mathbf{r}} = \delta_{\mathbf{kk'}} } $$

The integration runs over the entire mode volume *V*. If the field is tightly confined, i.e., it has a small mode volume, the modulus of **u**_**k**_ must be large enough to ensure the unity of inner product of **u**_**k**_ and u*_k_. Then Im[**G** (**r**, **r**, *ω*)] becomes considerable according to Eq. (2) and so is the Purcell factor according to Eq. (1). Thus, strong confinement of radiated fields is vital to SER enhancement, which inspires us to design metamaterials consisting of graphene.

**Fig. 1:**
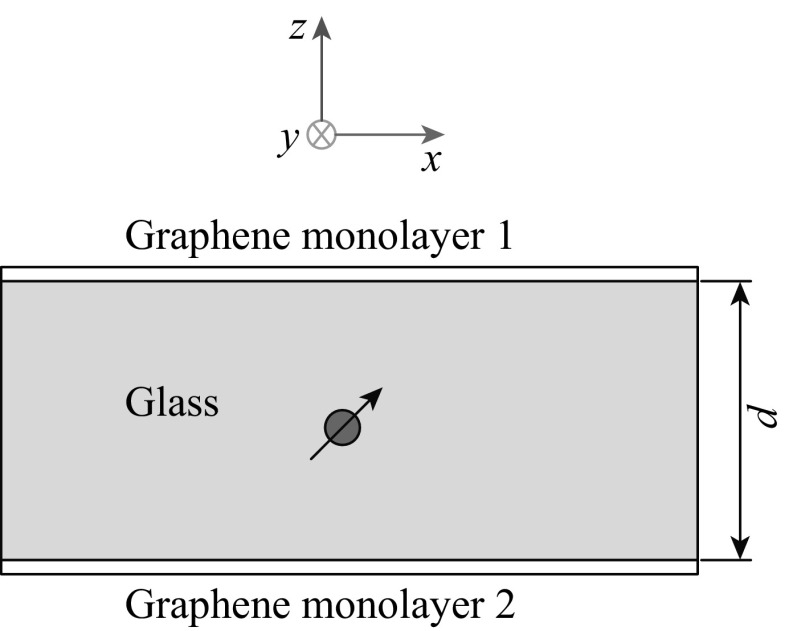
Schematic diagram of an ultra-thin glass film coated with graphene monolayer 1 and monolayer 2. Quantum emitter which is approximated by an electric dipole is placed at the center of glass film whose thickness is denoted as *d*

We propose a new kind of metamaterial whose structure is illustrated in Fig. 1. The thickness of the glass film *d* is usually on the order of tens of nanometers. Both the upper and lower facets of the film are covered with doped graphene monolayers. Glass is chosen as the middle layer for three reasons: (1) glass is transparent for MIR light and has low intrinsic losses in this regime; (2) graphene can be transferred to both sides of the glass layer by standard poly(methyl methacrylate) (PMMA) method [17]; (3) it also works as a supporting layer due to the ideal mechanical properties of glass. The size of structure in *x* and *y* direction is considerably large compared to the film thickness *d* (the ratio of film thickness to graphene monolayer length in the *x* direction is exaggerated in Fig. 1). The dipole emitter is placed at the center of the glass and its radiated fields are well confined by the extended graphene monolayers. The configuration of the proposed metamaterial is quite simple, which means its fabrication process is much easier than that of double-layer graphene waveguide reported in Ref. [12].

**Fig. 2:**
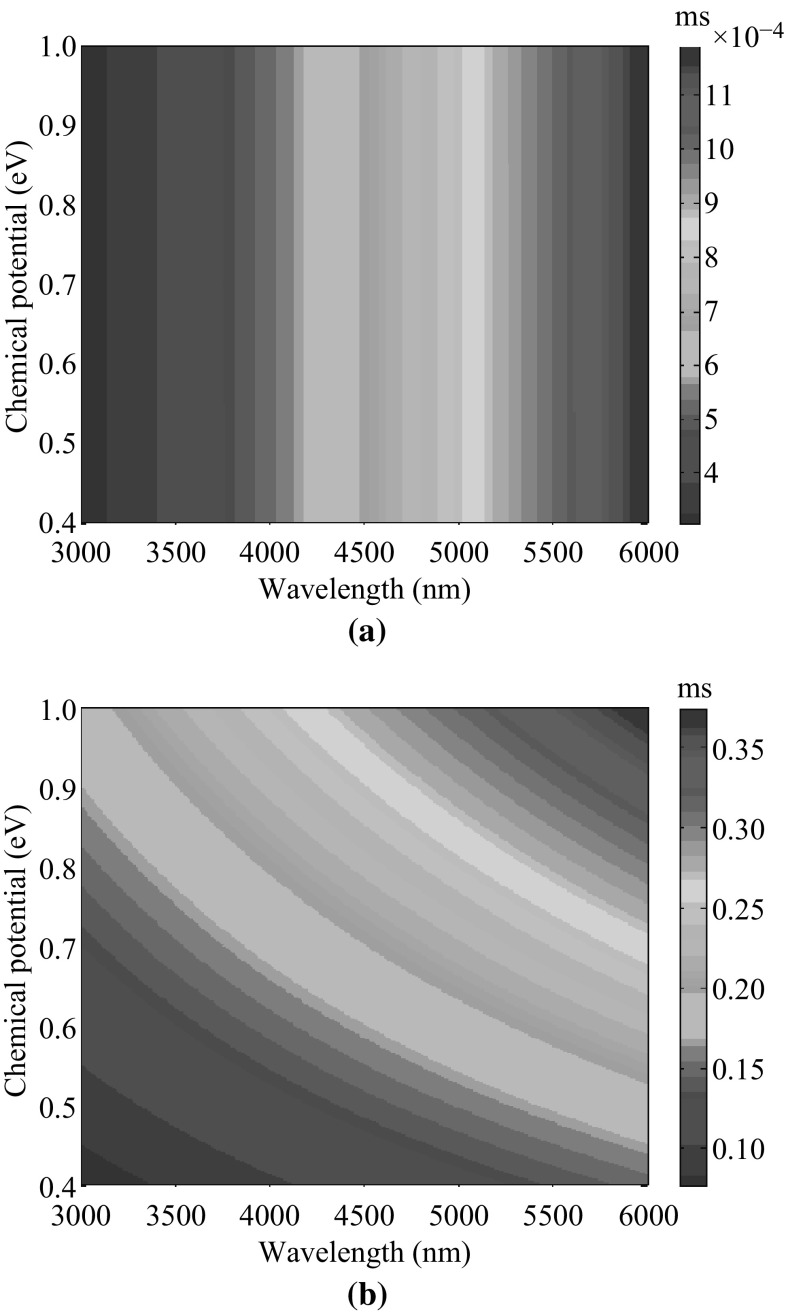
**a** Real part and **b** imaginary part of the conductivity of graphene for various chemical potentials (0.4–1 eV) and wavelengths (3000–6000 nm) in MIR regime

For MIR wavelengths considered here, the conductivity of graphene is dominated by intraband transition and can be simplified to a Drude-like expression as [18, 19]:

4 $$ \sigma_{\text{g}} = \frac{{i\mu_{\text{c}} e^{2} }}{{\pi \hbar^{2} (\omega + i\tau^{ - 1} )}} $$

where *e* is the charge of an electron, $\hbar$ is the reduced Planck’s constant, *ω* is radian frequency, and *τ* is electron relaxation time. The chemical potential *μ*_c_ can be tuned by varying chemical doping levels during the fabrication, i.e., $\mu_{\text{c}} = \hbar v_{\text{f}} \sqrt {\pi n_{g} } ,$ where *n*_g_ is the carrier concentration of graphene and $v_{\text{f}} \approx 10^{6}\,\text{m/s}$ is the Fermi velocity in graphene. The mobility of graphene *μ* is set to 10,000 cm^2^/(V s) which is a moderate value obtained in experiments [20]. It is related to electron relaxation time by $\tau = \mu \mu_{\text{c}} /ev_{\text{f}}^{2} .$ According to Eq. (4), the conductivity of graphene is calculated and plotted in Fig. 2a and b. The real part of conductivity is almost independent of chemical potential and increases monotonically with increasing wavelength while the imaginary part increases monotonically with increasing chemical potential and/or wavelength. Since $\text{Im} (\sigma_{\text{g}} ) > 0,$ only transverse-magnetic (TM) SPs are supported by graphene [21, 22]. They are supposed to be the main decay channels for quantum emitter discussed here.

## Numerical Results and Discussion

**Fig. 3:**
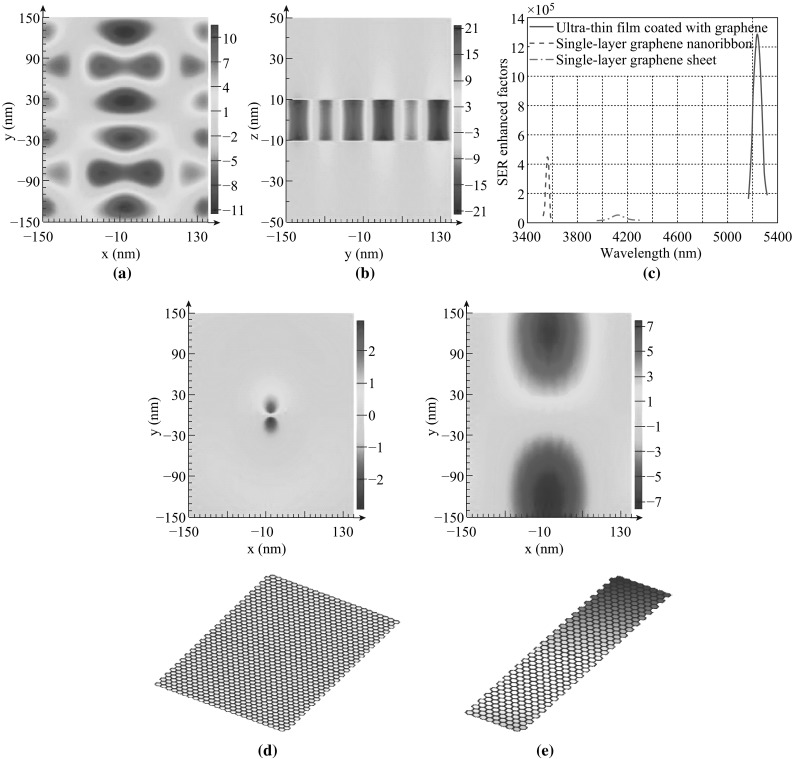
**a** *H*_*x*_ component in a graphene monolayer and **b** the *YOZ* plane of ultra-thin glass film coated with graphene. The film thickness *d* = 20 nm, dipole moment is orientated in the *z* direction, and chemical potential of graphene *μ*_c_ = 0.64 eV. **c** SER-enhanced factors of electric dipole near ultra-thin glass film coated with graphene (*blue solid line*), single-layer graphene sheet (*green dash-dotted line*), and single-layer graphene nanoribbon (*red dashed line*) of 100 nm in width. Schematics and *H*_*x*_ field distributions of single-layer graphene **d** sheet and **e** nanoribbon. Dipoles are placed 10 nm away from graphene monolayers and the chemical potentials of graphene are *μ*_c_ = 0.64 eV. (Color figure online)

To further investigate the nature of SER enhancement in the new material, we use FDTD techniques to calculate the electromagnetic field distribution in the proposed structure. The related parameters are set as follows: glass film thickness *d* = 20 nm, dipole moment oriented in the *z* direction, chemical potential of graphene *μ*_c_ = 0.64 eV. *H*_*x*_ components in graphene monolayer 1 and monolayer 2 have the same field pattern (Fig. 3a). As we can see, the energy of electric dipole is efficiently coupled into TM SPs of graphene, which is one of the most important decay channels in the near field. The field pattern of *H*_*x*_ component in the *YOZ* plane is shown in Fig. 3b. It is obvious that electromagnetic fields are confined between two graphene monolayers, i.e., inside the glass film. The SER-enhanced factors can be calculated by $\gamma /\gamma_{0} = P/P_{0},$ where *P* is the power radiated by electric dipole in surrounding material and *P*_0_ is the one in free space. We conduct all the numerical calculations using commercial software called Lumerical FDTD solution. The total power radiated by the dipole is calculated by measuring the power flow out of a small volume which contains the source. In our simulations, we set six power monitors on the faces of a box surrounding the dipole. We find the highest SER-enhanced factor peak in the MIR regime and compare it with those of single-layer graphene sheet and nanoribbon. The results are shown in Fig. 3c. Figure 3d and e shows the schematics of single-layer graphene sheet and nanoribbon and the corresponding *H*_*x*_ field distributions in them. The nanoribbon width is set to 100 nm. Dipole emitters are all placed 10 nm away from graphene monolayers. The chemical potentials of the single-layer graphene sheet and nanoribbon are both *μ*_c_ = 0.64 eV which corresponds to a doping density of 3.0 × 10^17^ m^−3^. The SER-enhanced factor of ultra-thin glass film coated with graphene reaches its maximum of 1.286 × 10^6^ at 5243 nm, which is about three times and 12 times larger than that of single-layer nanoribbon and single-layer sheet, respectively. We can also see that the magnitude of *H*_*x*_ field in ultra-thin glass film coated with graphene is much stronger than those in single-layer graphene sheet and nanoribbon. These results confirm the theories given in Sect. 2 that confinement of radiated fields is of great importance for SER enhancement, considering that ultra-thin glass film coated with graphene has better radiation field confinement than single-layer graphene sheet and nanoribbon.

**Fig. 4:**
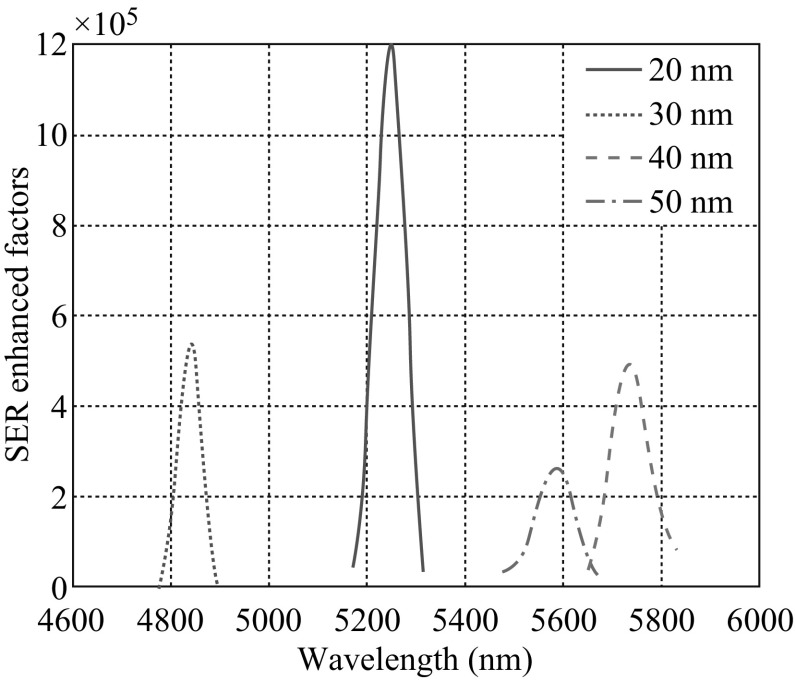
SER-enhanced factors for glass film thickness of 20 (*blue solid line*), 30 (*red dashed line*), 40 (*green dotted line*), and 50 nm (*purple dash-dotted line*). (Color figure online)

Next we investigate the influence of glass film thickness on SER-enhanced factor. The film thickness varies from 20 to 50 nm with a step of 10 nm. Other parameters are kept the same as in Fig. 3. The maximum SER-enhanced factor decreases monotonically with increasing film thickness (Fig. 4). This phenomenon can be explained as follows: with larger separation distance between graphene double layers, the radiated fields are less confined, which leads to weaker light-matter interactions. For film thickness less than 20 nm, the SER enhancement will be stronger. However, the complexity of fabrication will increase enormously. So we do not discuss thinner film thicknesses in this paper.

**Fig. 5:**
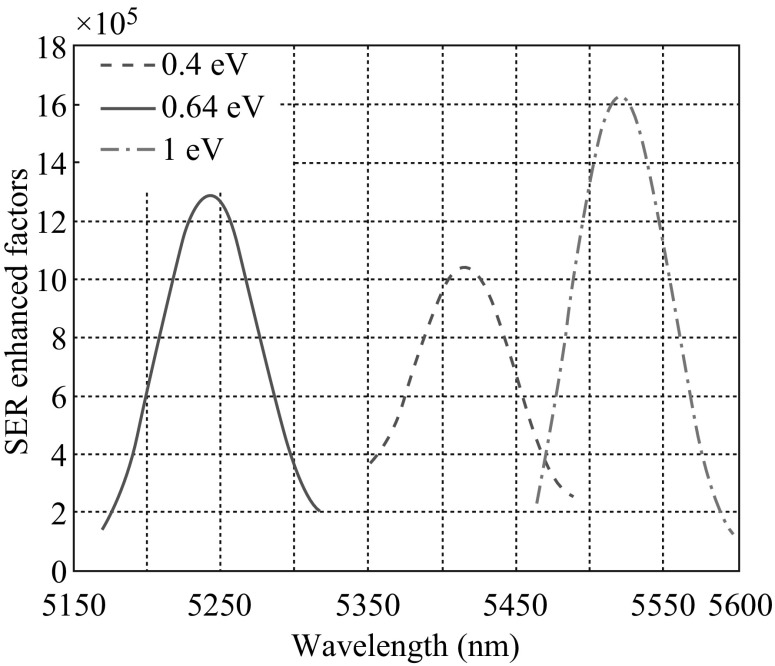
SER-enhanced factors for chemical potential of 0.4 (*red dashed line*), 0.64 (*blue solid line*), and 1.0 eV (*green dash-dotted line*). (Color figure online)

Another parameter which will affect the SER-enhanced factor is the doping level or chemical potential of graphene. The chemical potentials of 0.4, 0.64, and 1 eV which correspond respectively to doping densities of 1.17 × 10^17^, 3 × 10^17^, and 7.32 × 10^17^ m^−3^ are explored in this paper. Chemical potential lower than 0.4 eV is unconcerned in our discussion because in that case interband transition occurs in graphene with incident photons of MIR wavelengths and brings high losses to graphene SPs. The SER-enhanced factors are numerically calculated using FDTD techniques and the results are shown in Fig. 5. It is obvious that the metamaterial made of graphene with a higher chemical potential or doping level acquires a larger SER-enhanced factor. It can be explained considering the nature of graphene TM SPs whose propagation constant *q* and out-of-plane decay coefficients *Q*_1_ *, Q*_2_ can be expressed as [7]:

5 $$ q \approx Q_{1} \approx Q_{2} \approx \varepsilon_{0} (\varepsilon_{\text{r}1} + \varepsilon_{\text{r}2} )\frac{i\omega }{\sigma (\omega )} $$

for $q \gg \omega /c$ *ε*_1_ and *ε*_2_ are the dielectric constants of surrounding medium 1 and medium 2, respectively. *Q*_1_ and *Q*_2_ are the corresponding decay coefficients. Since the imaginary part of the conductivity of graphene is several orders larger than its real part, only the imaginary part needs to be considered in Eq. (5). The three resonance wavelengths in Fig. 5 are close to each other in the spectrum, so the imaginary part of conductivity mainly depends on the chemical potential of graphene (see Fig. 2). They are 0.1466, 0.2097, and 0.3450 mS which correspond to decay coefficients of 5.15 × 10^7^, 3.72 × 10^7^, and 2.15 × 10^7^ m^−1^ for 0.4, 0.64, and 1 eV, respectively. The fields of graphene TM SPs decay exponentially along the *y* direction. A higher decay coefficient will lead to a weaker interaction between the graphene and electric dipole placed 10 nm away from it. Thus, we can conclude that the dipole energy is coupled more efficiently to graphene with a higher chemical potential, which results in a larger SER-enhanced factor. This fact explains the phenomenon observed in Fig. 5.

The proposed metamaterial also enjoys high flexibility in several ways. For example, the frequencies at which SERs are enhanced can be selected by either tuning the chemical potential of graphene *μ*_c_ or changing the glass film thickness *d*, as indicated in Figs. 4 and 5. As for applications, the metamaterial can be directly integrated with quantum emitters as the surrounding medium to form highly efficient subwavelength single-photon sources.

However, there exist some factors that limit the SER-enhanced factor. Chemical doping will inevitably introduce scattering source and thus degrade the mobility of intrinsic graphene [23]. Meanwhile, supporting substrate will also bring additional scattering mechanisms such as interface impurity scattering and remote phonon scattering [24]. These cause the degradation of graphene mobility as well. For lower mobility, e.g., *μ* = 5000 cm^2^/(V s), an SER-enhanced factor of 1.197 × 10^6^ can be achieved in the structure. So in reality, the SER-enhanced factor will be lower than that obtained without taking mobility degradation into consideration, but still on the order of 10^6^.

**Fig. 6:**
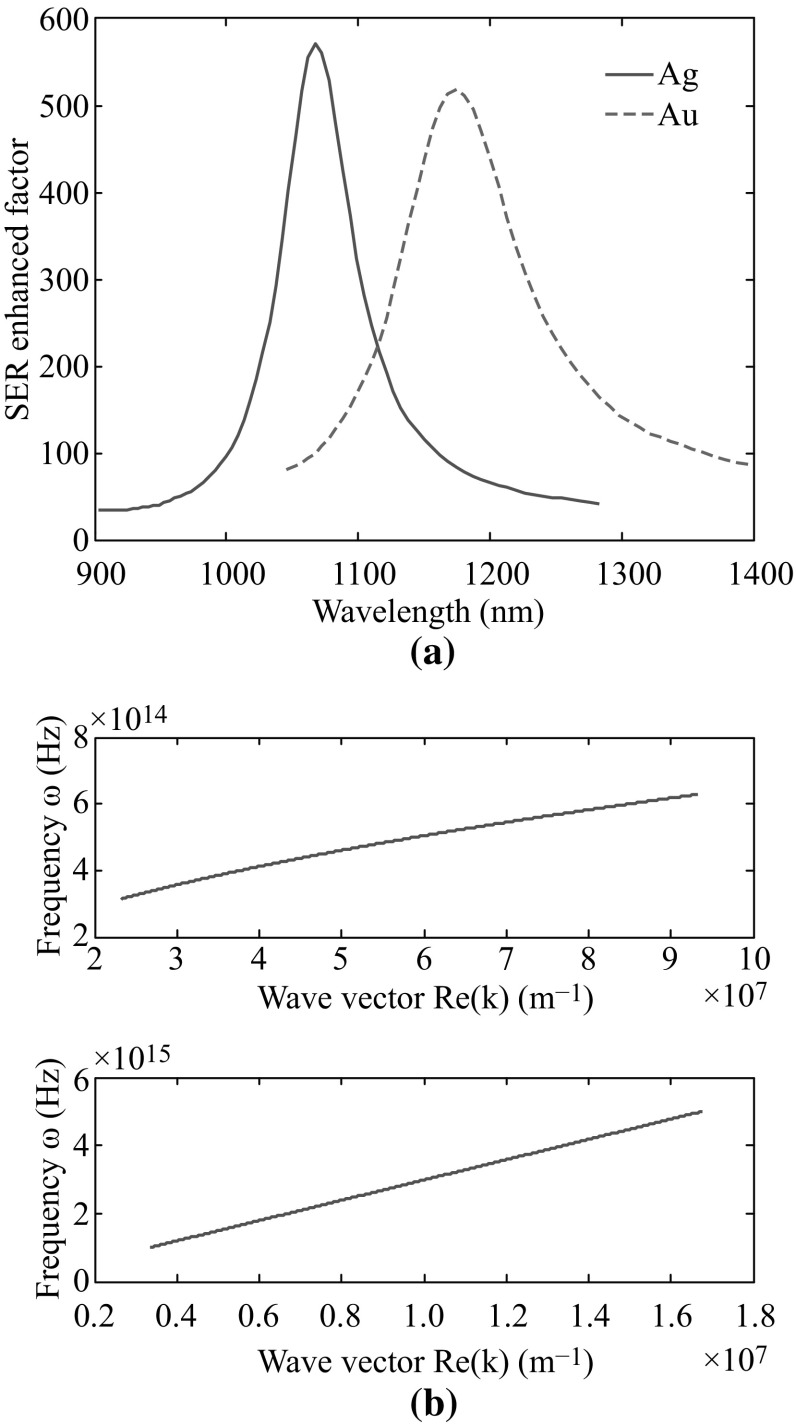
**a** SER-enhanced factors for gold (*red dashed line*) and silver (*blue solid line*) coatings, **b** dispersion relationships of TM SPs on silica–graphene and silica–silver interfaces. (Color figure online)

Finally, we compare graphene with other coating materials such as gold and silver. Two graphene monolayers shown in Fig. 1 are now replaced by 20 nm-thick gold/silver films. The maximum SER-enhanced factors of gold and silver coatings are given in Fig. 6a. The SER-enhanced factor of silver coating is a little higher than that of gold one due to its lower intrinsic losses at optical frequencies. Although SER-enhanced factors of over 500 can be achieved by both coating materials, they are still several orders lower than that of graphene. The reasons for the extremely high SER-enhanced factor of graphene-based metamaterial can be figured out after studying the photonic density of states of graphene TM SPs. The plasmon density of states $\rho (\hbar \omega )$ can be written as [25].

6 $$ \rho (\hbar \omega ) = \frac{{L^{2} }}{4\pi }\frac{{{\text{d}}(k^{2} )}}{{{\text{d}}(\hbar \omega )}} $$

where *L* ^2^ is the in-plane quantization area, *k* is the in-plane wave vector of plasmon. The dispersion relationships of TM SPs on silica–silver and silica–graphene interfaces are plotted in Fig. 6b according to which the photonic density of states of graphene SPs is generally three orders larger than that of silver ones, i.e., $\rho_{\text{graphene}} /\rho_{\text{silver}} \sim 10^{3}$. It is in high agreement with the ratio between SER-enhanced factors of glass films covered by graphene and silver coatings, which can be predicted by the Fermi’s golden rule. Therefore, we have come to the conclusion that the dispersion relationship of graphene TM SPs contributes tremendously to plasmon density of states in the space, which leads to a super high SER-enhanced factor of ultra-thin glass film coated with graphene.

## Conclusion

In conclusion, we proposed an ultra-thin glass film coated with graphene where the SERs of embedded quantum emitter were multiplied by a factor typically on the order of 10^6^. The influences of glass film thickness and chemical potential/doping level of graphene on SER-enhanced factor were also studied. A comparison of graphene and other coating materials including gold and silver was made in terms of SER enhancement. Explanations and quantitative analysis were given to the observed phenomena. Different from previously reported works, the metamaterial we proposed benefits from the nature of graphene SPs. The radiated fields are better confined in the ultra-thin glass film coated with graphene, which gives rise to stronger light-matter interactions and higher SER-enhanced factors. It has great potential to work as the surrounding material of highly efficient quantum emitters for novel single-plasmon devices and quantum plasmonic applications in the near future.
